# Twelve Tips for running in-situ simulation during a Coronavirus pandemic

**DOI:** 10.15694/mep.2021.000015.1

**Published:** 2021-01-19

**Authors:** Jennifer Pollard, Danielle Jeffreys, Donald Irvine, Ian Thomas

**Affiliations:** 1 NHS Highland; 2 University of Aberdeen

**Keywords:** in-situ, simulation, pandemic, decision-making, transitions

## Abstract

This article was migrated. The article was marked as recommended.

The arrival of the coronavirus pandemic has caused massive disruption to medical education, with universities having to close and adopt new ways of teaching, ensuring social distancing as standard. Final year medical students from the University of Aberdeen graduated early and stepped up to start working as ‘Foundation interim Year 1 doctors’ (FiY1). With their final months of medical school and end of year examinations cancelled, we felt that an in-situ ward simulation would help them make that transition by giving them an opportunity to act up in a safe environment. Here we share our tips for designing and implementing an in-situ simulation aimed at junior doctors in the early stages of their training. We conclude by reflecting on what we have learnt and how we plan to take this method of simulation forward into future practice, once the pandemic is over.

## Introduction

Preparing final year medical students for transition into the working world is one of the most challenging aspects of medical education. Students and doctors often feel underprepared for these transitions ( Morgan and Cleave-Hogg, 2002; Brennan *et al.*, 2010; McGregor *et al.*, 2012; J. C. Illing *et al.*, 2013; Cleland *et al.*, 2016) and studies have looked at a variety of ways to remedy this ( Gordon, Darbyshire and Baker, 2012; Bamford *et al.*, 2018). Simulation is a useful adjunct to teaching, especially in non-technical skills ( Weller, 2004; Paskins and Peile, 2010; Pucher *et al.*, 2014; Gee, Morrissey and Hook, 2015; Harvey *et al.*, 2015; Thomas *et al.*, 2015; Cleland *et al.*, 2016). At University of Aberdeen, we were already running Patient Care Simulations (PCS) with a mannequin teaching assessment of acutely unwell patients, and a simulated ward experience with volunteer patients, focusing on non-technical skills (NTS).

The COVID-19 global pandemic and consequent government lockdown directive meant a premature end to medical school and early graduation for final year medical students. An in-situ experience, as the name suggests, embeds the experience in a working clinical environment. In Raigmore Hospital, Inverness, we had access to a four-bedded area with a number of side rooms, that is vacant and awaiting clinical allocation, which we were able to utilise.

The aim of this simulation was to help students make the transition to Foundation Year 1, whilst encompassing new challenges they may face in light of COVID-19. Transitions are stressful ( McGlynn *et al.* 2012; J.C. Illing *et al.* 2013; J. Illing *et al.* 2013; Cleland *et al.* 2016) and our department was keen to support junior doctors in stepping up, especially during this uncertain time.

Learning objectives for FiY1 in-situ simulation were:

• To develop NTS, such as prioritisation and decision-making
• To simulate management of a critically ill patient with appropriate escalation of care
• To expose participants to a simulated COVID-19 patient to improve recognition

The patient scenarios designed to achieve these learning outcomes and are detailed in Table 1.

**Table 1.** Patient scenarios, learning objectives and distractions employed during FiY1 in-situ simulation

Patient scenario	Learning Outcomes	Tasks to complete	Distractions deployed
Brief handover of patients to participant from ward nurse at start of simulation	To rapidly assimilate information from other team members	To ask appropriate handover questions correct prioritisation of patients	None
40-year-old admitted with RUQ pain from A&E, with a working diagnosis of biliary colic Poor pain control, NEWS=1 for tachycardia	Challenging a pre-conceived diagnosis (identify this is pancreatitis not biliary colic) based on blood results Having the confidence to prescribe adequate analgesia (IV morphine)Dealing with distractions	Focused history and examination Review and prescribe adequate analgesia Appropriate handling of bleep and interruptions	Bleep from biochemistry with D-dimer result for undiagnosed COVID patient (>2000ng/ml)
25-year-old patient admitted with abdominal pain, fever and dysuria. Patient keen to go home Urine culture in the community - current antibiotic regimen is not correct (resistant organism)	Choosing appropriate antibiotics Identifying penicillin allergy Communicating discharge decision with patient	Prescribe antibiotics using the hospital protocol (accessed via the intranet) Advise patient why they cannot go home	Phone call to doctor’s office from the bed manager asking about discharges
42-year-old patient admitted with gastroenteritis, becomes rapidly hypoxic overnight. CXR shows bilateral changes and if asked, this patient has broken lockdown rules and been exposed to COVID at a group party	Assessing and managing an acutely unwell patient Recognise signs and symptoms of COVID-19, including atypical presentation Identify the need for escalating care to senior using SBAR technique	ABCDE assessment and appropriate oxygen therapy Thorough assessment, assimilation of relevant blood results Escalation to senior	No distractions at this patient, although appreciation that the high d-dimer call from biochemistry earlier reflects severe COVID rather than a pulmonary embolism.

We conducted this simulation with 24 Foundation interim Year 1 doctors (FiY1). After a positive reception, the opportunity was extended to 26 Foundation Year 1 doctors (FY1) and 22 ‘junior middle grades’, encompassing Foundation Year 2 doctors (FY2), General Practice Speciality Trainees (GPST), Internal Medicine Trainees (IMT) and Clinical Development Fellows (CDF). Each session involved four faculty - three observing and debriefing participants and one acting as nurse confederate within the simulation. The observing faculty took turns rotating between observing and debriefing the participants, deploying distractions and playing the role of an auxiliary nurse.

Having run simulations for a total of 72 doctors, we would like to present our 12 tips for running a successful in-situ simulation during a pandemic.

## Preparation/pre-simulation tips

### Tip One: Adapting the environment and socially distancing

The usual high-tech simulated ward environment in our Clinical Skills Centre (CSC) was not accessible during the pandemic. The first priority during simulation design was the need to accommodate social distancing measures. The change in environment meant considerations regarding ward layout, faculty and participant safety had to be factored into planning. To allow safe social distancing in the 4 bedded area, we only had 2 working bed spaces placed diagonally opposite from each other. Careful scripting of the scenarios ensured we were able to incorporate a side room by crafting an undiagnosed COVID-19 patient scenario with an initial presentation of diarrhoea and vomiting. The use of the side room for this patient not only reflected real life but also enabled greater social distancing for all participants.

Previously, in our CSC, several faculty members observed participants from a small room behind a one-way mirror into the simulated ward. Having faculty in such proximity was no longer feasible, so we simplified the delivery with a socially distanced facilitator who shadowed the ward round, took notes and then gave feedback to the participant. Debriefing rooms were carefully organised to facilitate social distancing, and we had to adapt to delivering feedback whilst wearing facemasks.

### Tip Two: Adapting to the loss of simulated volunteer patients

Due to lockdown rules, we did not have access to our usual bank of simulated volunteer patients (VP’s), most of whom were over 70 or shielding. VP’s are members of the general public who portray a patient according to a careful script ( Barrow, 1993). The use of VP’s, as opposed to mannequins, has the advantage of providing a real patient interaction without subjecting real patients to potential mistreatment ( Wallace, Rao and Haslam, 2002).

To overcome the absence of VP’s, we had to get creative by asking experienced surgical and medical Registrars or Consultants to role-play patients who, with their clinical experience, could enact the scenarios effectively. Having participated in the first simulation, a number of the FiY1s were keen to be involved in simulations for more senior trainees. We utilised the FiY1 doctors not only as VP’s, but as simulated junior doctors and the benefits of this were two-fold. Firstly, the more senior trainees had an opportunity to delegate to and teach a more junior team member. Secondly, the FiY1 found it beneficial to watch more senior doctors going through the experience, enabling them to learn by ‘osmosis’ and benchmark their own performance.

### Tip Three: Having flexible faculty

With the limited technology of the in-situ ward, we also had to get creative with how best to use our faculty.

Previously, four faculty members would remotely observe from behind mirrored glass, carefully orchestrating the simulation. We were used to being in control via microphones and earpieces to ‘in room’ faculty and directing the scenario to achieve specific outcomes. With in-situ simulation, there are no mirrors or remote communications. As such, the participant is much more in control and you are reliant on your simulation design to ensure the learning objectives are met. Our faculty adapted easily to handing over the reins to participants and this new ‘organic’ version of the ward experience was positively received by both trainees and faculty.

Another new aspect for the faculty was having to assume other roles. This was important to allow social distancing, as we could no longer have faculty members grouped together in a small space watching the simulation. A good example of this was the auxiliary nurse role, scripted specifically for this purpose to take patient observations and comment that certain patients weren’t looking well. This role helped to socially distance faculty, boosted their engagement with varied roles and added to the fidelity of the experience.

### Tip Four: Embracing change

Initially, it was daunting for our team to consider functioning without the usual high-tech equipment and remote observation from behind mirrored glass. However, faculty embraced the simplified changes and were surprised to find they enjoyed being part of a more ‘organic’ process.

Despite pre-simulation reservations, faculty commented they felt more immersed in the method and gained enhanced perception by being present in the room. Faculty were able to pick up on non-verbal cues more readily, such as body tension when participants were overwhelmed, and observe the whole scenario from a much better vantage point than through a small window.

Faculty delivering distraction elements and answering the phone for senior advice also had reservations about not knowing where the participants were in the scenario without the benefit of a one-way mirror. However, they found simply reacting to the information communicated via the participant quite liberating and more realistic.

Our faculty demonstrated that willingness to embrace change that may initially appear uncomfortable had beneficial consequences, and this is relevant to how we will all need to adapt within the NHS in the post-COVID era.

### Tip Five: Patient scripting in a pandemic

Although the primary aim of the simulation was to aid transition from undergraduate to FiY1, we were also keen to expose participants to assessment and management of a patient with COVID-19, something not taught at medical school.

A scenario was created where a young patient was admitted with an initial presentation of diarrhoea and vomiting, reflecting 10% of COVID-19 patients who may present with gastrointestinal (GI) symptoms. The patient then developed sudden respiratory deterioration with classic COVID-19 symptoms of shortness of breath, cough and fever. Blood results confirmed leucopenia, high C-reactive protein (CRP) and raised d-dimer, along with an arterial blood gas reflecting significant type one respiratory failure. A side-story related to breaking lockdown rules and contact with infected people was included for participants to uncover, highlighting the importance of enquiring about potential coronavirus exposure.

This scenario was useful, not just in reflecting the current healthcare climate, but also presenting an acutely unwell, rapidly deteriorating patient. It allowed us to create learning objectives focusing on ABC assessment, escalation of care, telephone communication to a senior, and considering appropriate personal protective equipment (PPE) for the trainee and other ward staff.

We felt it was important to have an element of the pandemic incorporated into the story lines of all the simulated patients, not just the COVID-19 scenario. For example, a post-operative laparoscopic appendicectomy patient was reluctant to go home and get back to work, as they were a key worker. The patient had concerns about their risk of contracting coronavirus on returning to work as a supermarket delivery driver. This challenged participants to uncover patient concerns and encouraged them to begin developing clinical decision-making and discharge-planning skills. Careful scripting like this helped to immerse participants in the scenario we are all currently living through.

### Tip Six: Setting the scene

Another important element to simulation design was contemplating how we would present the ward environment to participants. We deliberately used the current pandemic hospital set-up and staged the scenarios within the ‘Green Zone’ of the hospital, where patients were triaged as low risk of having COVID-19. This introduced an element of surprise when one patient deteriorated with a new diagnosis of COVID-19, highlighting the importance of thinking on their feet and adapting to new information. The use of a real ward area further added to the realistic setting and allowed participants to immerse themselves in a more familiar environment.

## Intra-simulation tips

### Tip Seven: Maintaining realism

Realism is an essential aspect of simulation ( Ker *et al.*, 2006; Brigden and Dangerfield, 2008; Scalese, Obeso and Issenberg, 2008) and we used several tools to enhance the realism of our scenarios. The in-situ setting enabled us to make use of distractions, such as call bells, which added to the hustle and bustle alongside the usual mixture of phone calls, bleeps and other disturbances.

“felt much more realistic being on a ward”

“more accurate representation of clinical environment”

The realism of the current healthcare climate was augmented by ensuring participants and faculty wore PPE, in the form of surgical facemask, gloves and plastic aprons, from the beginning of the simulation. Asking participants to don PPE before coming into the ward to be briefed was a deliberate tactic to enhance the simulation credibility and also ensured we protected each other from potential spread.

By involving faculty in role-play, we were able to maintain the patient interaction elements and allow participants to become more immersed in the simulation. Faculty could also enhance their story if required, unlike VP’s who are asked to stick rigidly to a script.

The auxiliary nurse role was multifaceted as they were perfect for deploying distractions (such as hoovering or manning the tea trolley) but could also be used to direct participants to the deteriorating COVID-19 patient if they missed earlier cues. The advantage of using faculty in this role, rather than a lay person, was they were able to employ autonomy in titrating the level of distraction up or down, depending on subtle cues from the observer.

## Post-simulation tips

### Tip Eight: It’s all in the debrief

Relevant, focused feedback greatly enhances the learning ( Ende, 1983; Issenberg *et al.*, 2005; Hattie and Timperley, 2007; Salas *et al.*, 2008; Ahmed, 2014) and we used a modified Pendleton approach. After asking how they felt, to allow participants to de-stress, we asked them to volunteer what they felt went well/not so well before providing comments on observations. Phrases such as ‘this is what I saw that was really good...’ and ‘these are areas I thought you found challenging...’ allowed us to give non-judgemental, constructive feedback regarding observed behaviours. Participants were encouraged to consider how they would approach challenges going forward and formulate action plans for ongoing development.

Both faculty and trainees noted the added difficulty of communicating whilst wearing facemasks and how much non-verbal communication was consequently missing. Faculty compensated for that by using other tools to reinforce the message, such as summarising the action plan at the end of the debrief. It also made for thoughtful consideration for how our patients could be feeling on the real ward, given how difficult it was for us medical professionals.

In previous simulations, we have sought feedback from VP’s and nurse confederates on trainee performance. There is some uncertainty about the use of VP feedback, and there is reasonable variation in the ways feedback is delivered and the training they receive ( Bokken *et al.*, 2009). Some faculty had reservations about whether VP feedback impacts on the ‘reaction time’ of the debrief - those initial few minutes where participants are encouraged to ‘let off steam’ immediately following the end of simulation. However, others felt VP’s can provide a valuable perspective on participant performance, which can be less effective if delivered by a facilitator. A systematic review by Bokken *et al.* (2009) suggests that VP’s should be encouraged to provide feedback from the patient’s perspective as this best capitalises on the uniqueness of the feedback they can offer. The choice to seek this additional feedback is probably ultimately down to personal choice and was left to the discretion of on-the-day faculty.

### Tip Nine: High fidelity doesn’t need to equal high-tech or cost

Just because we *can* use technology to deliver simulation, doesn’t mean we necessarily *have* to.

**Figure 1.**
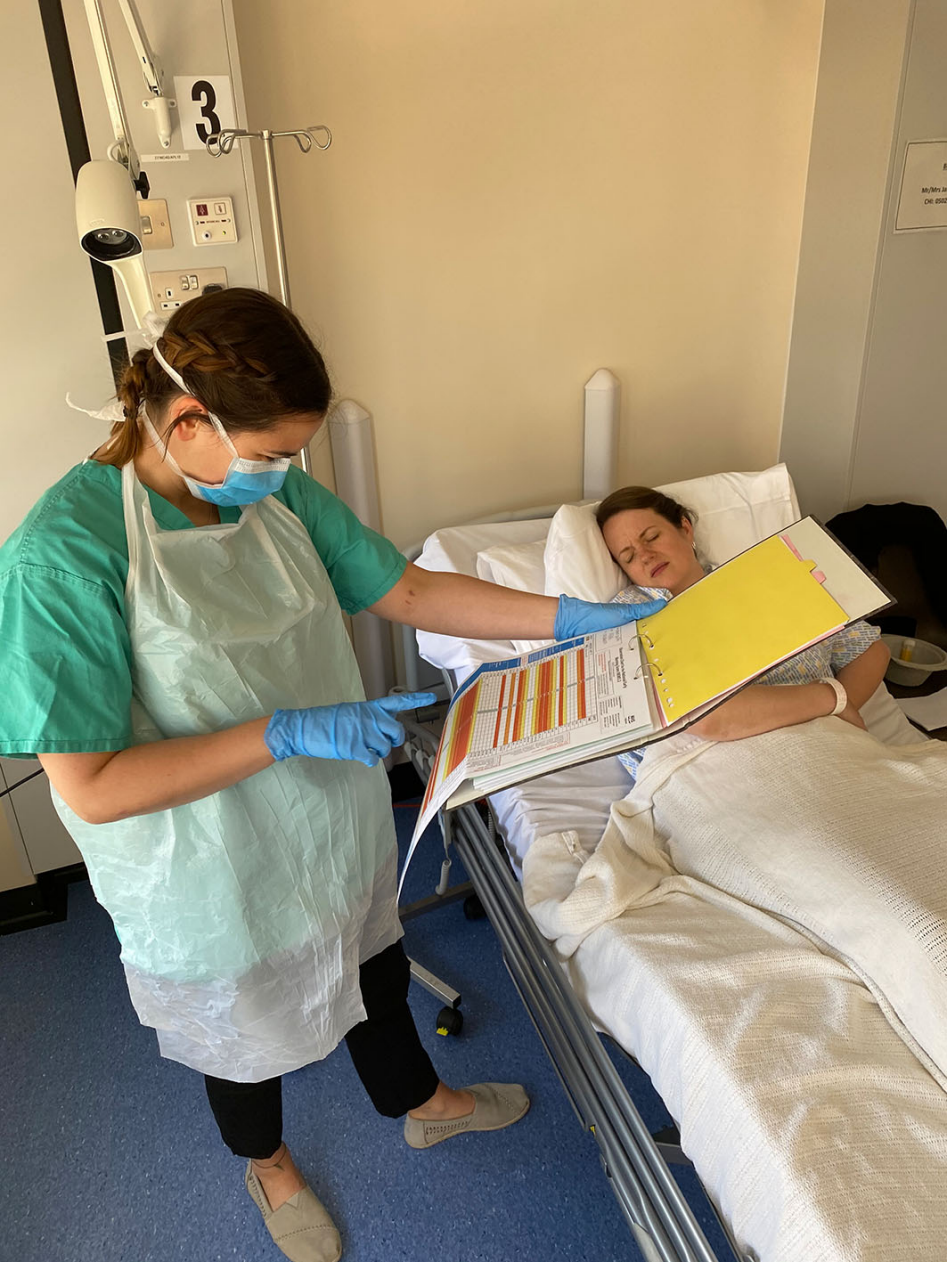
authentic paperwork to enhance the realism

For this in-situ simulation, we took the design back to basics. Replica admission folders were created for all simulated patients (including medical notes, drug charts, observation charts and referral letters) using photocopies of blank NHS Highland paperwork to enhance scenario authenticity ( Figure 1).

FiY1s had access to a ‘doctor’s office’ with an NHS computer connected to the hospital intranet. Documents that participants were expected to write on (e.g. prescription charts) were laminated to allow faculty to erase contents with alcohol wipes between simulations. This had the additional benefit of allowing us to recycle supplies and keep costs down.

Faculty also commented that they preferred this more ‘organic’ version of the ward round. They were able to immerse themselves in observation, rather than being distracted by using available technology.

Reflection post-simulation encouraged our faculty to consider negative influences of technology, and participant feedback confirmed that setting and realism of scenarios were more important than equipment. We also thought about whether the use of technology has overshadowed basic teaching methods employed in the clinical environment. This is relevant to the current pandemic as clinicians have been compelled to utilise more technology-based interactions with patients in the interests of social distancing and reducing spread of COVID-19. We think it will be essential to remember the lessons we have learnt from this when we return to normal.

### Tip Ten: Respond to feedback

Encouraging participant feedback was a key element in the development phase of the in-situ simulation, with suggestions of areas for improvement an ongoing process throughout simulation delivery. Participants were encouraged to provide either verbal or anonymous written feedback via questionnaire.

Participants generally felt the simulation was realistic. They provided insightful tips on what would enhance the realism further, which were acted upon in real time. For example, the addition of curtains around bed spaces and normal reference values supplied alongside blood results. Previously, curtains were not used in CSC as they obstruct the cameras used for observation. However, this was not an obstacle in the in-situ setting and participants commented that their presence augmented the experience.

Since our FiY1 doctors had been through simulated ward experiences as undergraduates, they were asked whether they felt running the simulation in-situ, as opposed to the CSC, added to the value of the simulation. The overwhelming majority reported that it was much more realistic and an accurate representation of the clinical environment. Some participants commented that the experience was similar in the two settings, however there were elements they preferred in-situ, such as having the assessor in the room rather than observing behind the mirror. This has caused us to consider the viability of developing a programme of in-situ simulations, even in the post-COVID era.

### Tip Eleven: Channel your artistic side

**Figure 2.**
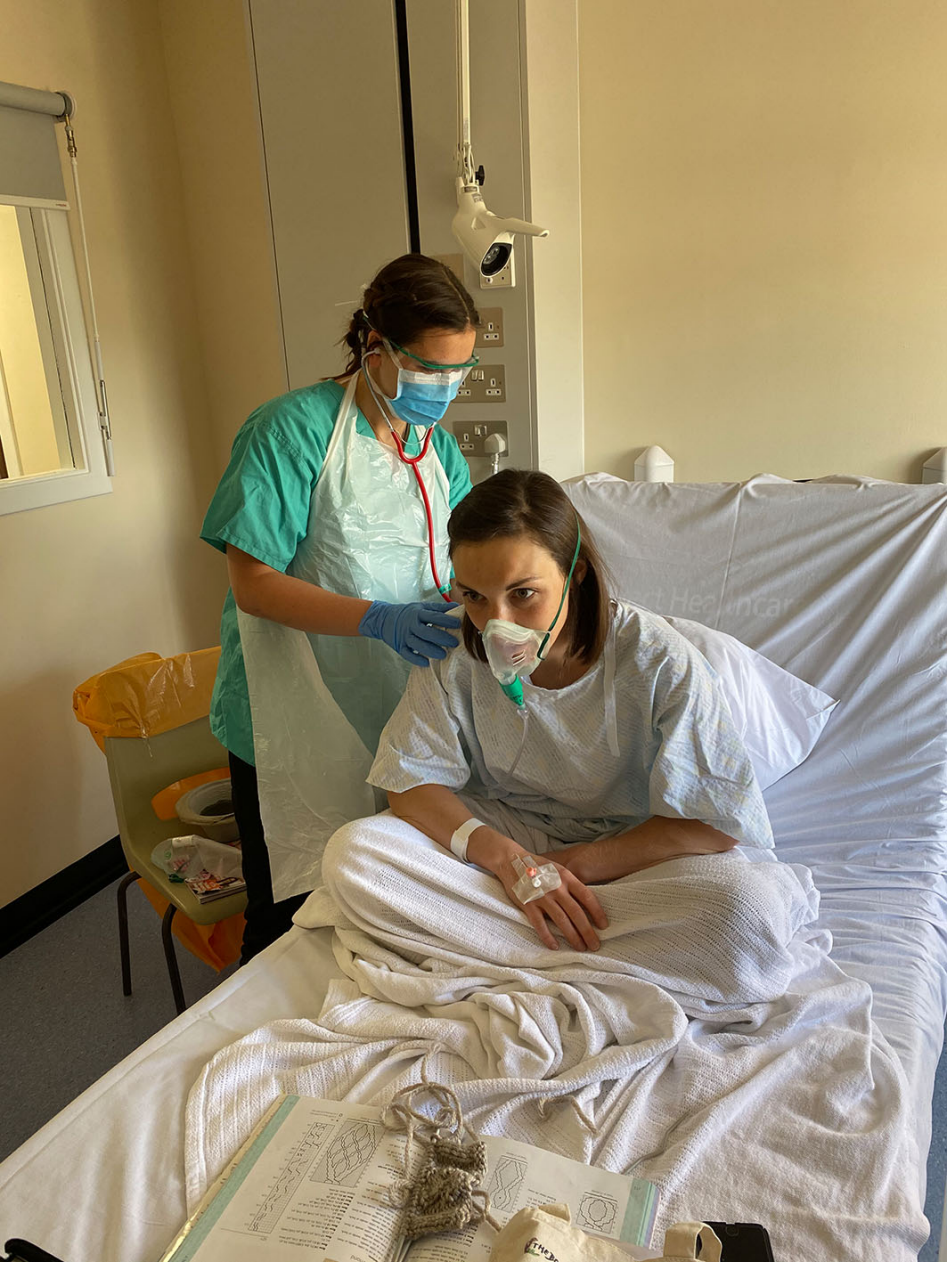
faculty member acting as COVID patient, with creative props

Using real people (as opposed to mannequins) allowed us to get creative with make-up, further adding to the realism of the scenarios, particularly with the COVID-19 patient. Careful attention was also given to the props in the room ( Figure 2). One participant stated:

“small details make it feel more realistic”

The initial presentation of the COVID-19 patient was with diarrhoea and vomiting. To replicate this, we used a sick bowl with green food colouring, water and flour to simulate bilious vomit. To mimic the patient’s respiratory distress, we used blue face paint to represent cyanotic lips and gelatine spray on the forehead to create the sweaty appearance associated with pyrexia.

The patient was already on 60% oxygen via Venturi mask, but there were appropriate devices in the room for escalating oxygen delivery, such as a non-rebreathe mask. As this was a real ward environment, participants were able to connect oxygen masks to the supply of oxygen in the wall but were asked not to turn on the flow.

**Figure 3.**
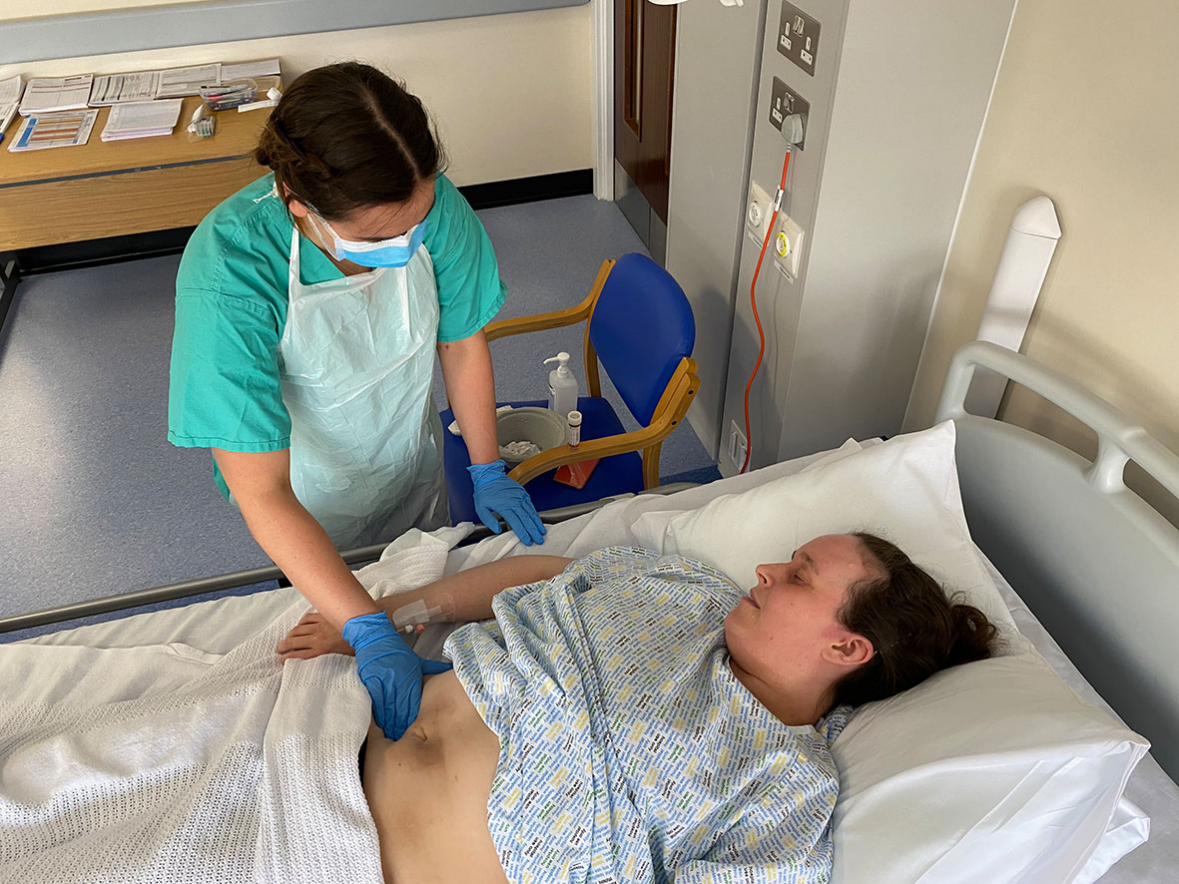
Mixed red and blue face paint on simulated patient’s abdomen to simulate brown bruising appearance of Cullen’s sign in acute pancreatitis

Mixed red and blue face paint was also used on an abdomen to simulate brown bruising appearance of Cullen’s sign in acute pancreatitis ( Figure 3).

All simulated patients were encouraged to leave belongings near their bed and bring a book to represent the usual paraphernalia patients have at their bedside.

### Tip Twelve: Create multi-dimensional patient scenarios

The scenarios can be easily adapted to cover a range of curriculum requirements for doctors of increasing seniority. The COVID-19 patient was more than just an opportunity to develop skills surrounding recognition, management and escalation of a deteriorating patient. For example, an important Foundation Year 2 (FY2) competency is being able to challenge the behaviours of others ( UK Foundation Programme Office, 2016; General Medical Council, 2017), which is a difficult element of the curriculum to gain real-life exposure and receive feedback on. Therefore, for the FY1 in-situ simulation, we carefully scripted an opportunity to explore this area. Once the participant had fully appreciated the suspected COVID-19 diagnosis, the auxiliary nurse entered the room with a tea-trolley to offer refreshments to the patient but was not wearing any PPE. This encouraged participants to be mindful of the behaviour of colleagues and to feel confident challenging them.

Many participants found this difficult and the debrief provided an opportunity to discuss tools for graded escalation of concerns to help them with future experiences. For example the CUSS ( **C**oncern, **U**ncomfortable, un **S**afe, **S**top) or PACE mnemonics ( **P**robe, **A**lert, **C**hallenge, **E**mergency action) ( Iedema, Piper and Manidis, 2015; Royal College of Paediatrics and Child Health, 2018) providing a structure for team members to reduce the chance of conflict whilst questioning the actions of others.

## Conclusion

By utilising the 12 tips outlined above, we have demonstrated we can still deliver high fidelity simulation training, without the need for high-tech equipment. We have demonstrated commitment from faculty in maintaining education and training considering the global pandemic, adapting to work with our new circumstances whilst maximising the learning experiences of our junior doctors.

Modifying our approach has caused us to evaluate the high-tech version of the simulated ward experience for our undergraduates, when we resume full educational activity. Important lessons have been learned in what is essential to ensure an effective learning experience versus what is being used simply because it is available.

The development of this in-situ simulation has been a learning experience for all involved, not just participants. Both faculty and trainees have had to evaluate the impact of a loss of non-verbal communication as a result of necessary PPE precautions surrounding COVID-19. We are all more mindful of patient interactions and how to ensure good communication is not hampered by these alterations to practice.

Going forward, we are planning to offer in-situ simulations for trainees at other transition points in their training, such as senior registrars approaching Consultant level. This will capitalise on an environment of change with a cohort of medical professionals who are receptive to rapid reshaping of the working environment.

## Take Home Messages

• flexible faculty are key to redesigning any simulation in an in-situ setting
• high fidelity doesn’t necessarily mean high tech or cost
• careful scripting can help immerse participants in the scenarios
• remember the importance of the debrief on learning outcomes
• don’t be afraid to get creative to enhance the realism

## Notes On Contributors

Jennifer Pollard is a speciality trainee in General Surgery and former University of Aberdeen Clinical Teaching Fellow. She has a Masters in Medical Education and is interested in simulation-based education and non-technical skills.

Danielle Jeffreys is a specialist trainee in Respiratory Medicine and is currently out of programme as a Clinical Teaching Fellow with the University of Aberdeen. She is currently working on a Diploma in Clinical Education. Her academic interests are mostly involved with transition from medical school to the working world.

Donald Irvine is a specialty trainee in Anaesthetics and a current Clinical Teaching Fellow with the University of Aberdeen. He is currently studying towards his Postgraduate Certificate in Education and has interests in medical innovation and technology.

Ian Thomas is a Consultant General Surgeon and Associate Director of Medical Education with NHS Highland, Inverness. He has a Masters in Medical Education with a research interest in simulated ward rounds and patient safety.
