# Supplementary material for: Twelve Tips for running in-situ simulation during a Coronavirus pandemic
Source: MedEdPublish (2016). 2021 Jan 19;10:15. [Version 1] doi: 10.15694/mep.2021.000015.1 (PMC10939556; doi:10.15694/mep.2021.000015.1)
Supplement: Peer review history

# Reviewer report: Reviewer response for version 1

**DOI:** 10.21956/mep.18836.r26835

**Published:** 2021-02-02

**Authors:** Johnny Lyon Maris

**Affiliations:** 1 Southampton GP Education Unit

This review has been migrated. The reviewer awarded 4 stars out of 5 Thankyou for sharing your experiences, it is important that as educators practical designs are shared as openly as possible for us all to learn. The statement for me that is the most important is 'high fidelity doesn't necessarily mean high tech or cost'. You have shown that cutting the simulation back to its component parts and simplifying it has gained feedback about reality. Simple touches like leaving the patient's bits and bobs lying around, give the appearance of reality.There has been a move away from health professionals role-playing patients, as it has been stated they either over or under-play the role. While this is important for assessment purposes (where consistency is key) for development purposes I am glad you have found it beneficial, with 2 way learning being an unexpected outcome.I think there is place for more photos to allow for key messages to be delivered. The ones included were helpful.

Reviewer Expertise:

NA

No decision status is available

# Reviewer report: Reviewer response for version 1

**DOI:** 10.21956/mep.18836.r26836

**Published:** 2021-01-21

**Authors:** Subha Ramani

**Affiliations:** 1 Harvard Medical School, Brigham and Women's Hospital

This review has been migrated. The reviewer awarded 3 stars out of 5 This is a timely 12 tips articles on developing a simulation program for assessment of clinical trainees. The authors have listed the tips in a logical order that will help simulation experts go through the design step by step. Thea article is well written, well referenced and the expertise of the authors is evident.The one comment I have is the imbalance in the number of tips- more than half in the pre-simulation phase and only one in the intrasimuljation phase. I wonder if tips 3 and 4 are that significantly different, for example.Under intra-simulation tips, I agree with realism but feel an additional tip on faculty behavior might be useful.Tips 1 and 2 start with adapt, perhaps 1 could be more specific in referring to the physical environment. Is "loss" of simulated patients the right word?In any case, this is a very useful article to all educators who design simulation based programs- assessment and otherwise. I also see these tips being useful beyond clinical skills assessment scenarios.

Reviewer Expertise:

NA

No decision status is available
